# Relationship between Permanent Catheter Patency and Nutrient Score in Patients Aged >75 Years Requiring Renal Replacement Therapy

**DOI:** 10.3390/jcm13061562

**Published:** 2024-03-08

**Authors:** Moo Jun Kim, Yunkyeong Hwang, Jae Wan Jeon, Hae Ri Kim, Suyeon Han, Heewon Park, Eu Jin Lee, Young Rok Ham, Ki Ryang Na, Hyerim Park, Dae Eun Choi

**Affiliations:** 1 Department of Nephrology, Chungnam National University Sejong Hospital, Sejong 30099, Republic of Korea; kimmoojun@cnuh.co.kr (M.J.K.); jeonjwan@cnuh.co.kr (J.W.J.); yo0118@cnuh.co.kr (H.R.K.); 2 Department of Nephrology, Chungnam National University Hospital, Daejeon 35015, Republic of Korea; hyk5210@gmail.com (Y.H.); garlic1208@cnuh.co.kr (S.H.); heewon910125@gmail.com (H.P.); eujinlee@cnuh.co.kr (E.J.L.); youngrok01@cnuh.co.kr (Y.R.H.); drngr@cnu.ac.kr (K.R.N.); 3 Department of Medical Science, Chungnam National University, Daejeon 35015, Republic of Korea; hye05240@gmail.com

**Keywords:** hemodialysis, permanent catheter, patency, nutrient factors, CONUT score, GNRI score

## Abstract

**Background:** Malnutrition is common in patients undergoing hemodialysis and is a powerful predictor of morbidity and mortality. This study aimed to investigate the effect of nutritional status on permanent catheter patency in elderly patients aged >75 years of age undergoing dialysis using tunneled dialysis catheters; **Methods:** Records of 383 patients whose nutritional factors and body cell mass (BCM) were measured simultaneously at the start of dialysis between 14 January 2020 and 30 September 2023, at Chungnam National University Hospital, were retrospectively reviewed. The relationships between permanent catheter patency at 180 days and BCM parameters and clinical parameters were studied using Kaplan–Meier survival curves and multivariate Cox proportional hazards analysis. **Results:** Age and sexual differences were significant (*p* ≤ 0.05), and most of the BCM parameters and BCM were not significant (*p* ≤ 0.05), except for intracellular water. Permanent catheter patency was superior at low controlling nutritional status (CONUT) scores (*p* < 0.05). After adjustment for covariates, the CONUT score remained an independent factor associated with permanent catheter-patency survival; **Conclusions**: CONUT scores measured before the start of dialysis are expected to play an important role in predicting the prognosis of permanent catheter-patency survival in patients aged >75 years.

## 1. Introduction

The number of older patients with end-stage renal disease (ESRD) is increasing, and a rising trend in the initiation of dialysis was also noted in this population [1]. In 2025, according to Statistics Korea, South Korea is predicted to transition into a superaged society. Along with this aging trend, the average age of patients undergoing dialysis increased to 65.0 years in 2019 [2]. Initiating dialysis in older patients with a heightened burden of age-related issues raises various concerns, including the choice of vascular access. In addition, some studies have suggested that due to the high failure rates in arteriovenous fistula (AVF) maturation and the relatively low infection rate of tunneled dialysis catheters, maintaining tunneled dialysis catheters in the long term could be considered a possible approach [3]. Nutritional status is an important issue in patients undergoing hemodialysis (HD). Malnutrition is common in patients undergoing HD and is a powerful predictor of morbidity and mortality [4,5]. The controlling nutritional status (CONUT) score is associated with all-cause mortality and predicts the initiation of dialysis in patients with chronic kidney disease [6]. The geriatric nutritional risk index (GNRI) is a predictive marker of malnutrition. Patients with a low GNRI score were associated with a higher utilization rate of tunneled dialysis catheters, and their survival rates were significantly lower [7]. HD is accompanied by catabolic and inflammatory processes that result in protein-energy wasting (PEW) [8,9]. When hemodialysis is required in the older population, the insertion of a dialysis catheter is necessary. In this situation, the patency of the catheter may persist for a long time, and in other cases, malfunctions may occur within a short period of time depending on the individual. However, few studies have attempted to clarify the predictors of survival of permanent catheter patency in hemodialysis patients, particularly in patients older than 75 years. And studies on the patency of permanent catheter and nutritional status targeting only the older population have not yet been actively conducted yet. It is unclear whether nutritional status affects the patency of tunneled dialysis catheters when their use becomes unavoidable in the older population. Therefore, this study aimed to investigate the effect of nutritional status on permanent catheter patency in aged >75 years undergoing dialysis with tunneled dialysis catheters because of the limited evidence for this association. In addition, this study aimed to investigate whether BCM parameters have a major effect on patients aged >75 years undergoing dialysis. 

## 2. Materials and Methods

### 2.1. Study Design and Participants

**Figure 1:**
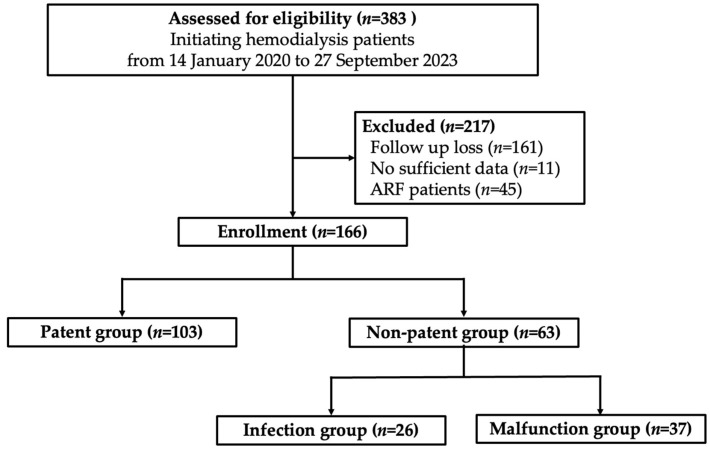
Study design flow chart.

The records of 383 patients whose nutritional factors and BCM were measured simultaneously at the initiation of dialysis between 14 January 2020 and 30 September 2023, at Chungnam National University Hospital, were retrospectively reviewed. Patients aged >75 years were eligible for inclusion in the study, and among them, the study targeted patients who had undergone blood sampling and body-composition tests in advance for renal replacement therapy. Of the 383 patients, 217 were excluded for the following reasons: lost during follow-up (*n* = 161), insufficient data (*n* = 11), and acute renal failure (ARF; *n* = 45) that improved after transient HD and stopped dialysis shortly after. Of 166 eligible patients, those who continuously used the permanent catheter without abnormalities were designated as the patent group (*n* = 103). Patients who had problems using the permanent catheter within 180 days were assigned to the nonpatent group (*n* = 63). Patients with confirmed catheter infections were subclassified into the infection group (*n* = 26), and those whose HD was not performed normally through the catheter were subclassed into the malfunction group (*n* = 37) (Figure 1).

Information collected from the medical records review included sex, age, duration of renal replacement therapy through the dialysis catheter (vintage day), underlying disease (hypertension [HTN], diabetes mellitus [DM], cardiovascular disease, cerebral infarction, and liver cirrhosis), ESRD cause, medication history (aspirin, clopidogrel, warfarin, cilostazol, nonvitamin K antagonist oral anticoagulants, and statin), catheter insertion date, catheter follow-up management, reason for catheter removal, catheter removal date, catheter usage period, BCM parameters (extracellular water [ECW], intracellular water [ICW], total body water [TBW], ECW/ICW ratio, lean tissue index [LTI], fat-tissue index [FTI], lean-tissue mass [LTM], adipose-tissue mass [ATM], fat mass [FAT], dry weight, and body mass index [BMI]), laboratory tests (hemoglobin, platelet, total lymphocyte count, C-reactive protein, total protein, serum albumin, blood urea nitrogen, creatinine, total calcium, phosphate, sodium, and potassium), pre-HD weight, post-HD weight, and ultrafiltration. 

This study was conducted in accordance with the ethical standards laid down in the 1964 Declaration of Helsinki and its later amendments. The medical records of the patients were retrieved anonymously in accordance with data-protection rules after obtaining ethical approval from the ethics committee of the hospital. This study was conducted according to the guidelines of the Declaration of Helsinki, and approved by the international review board of Chungnam National University Hospital (IRB No. 2023-11-078 date of approval 9 January 2024). 

### 2.2. Definitions

The nonpatency of a permanent catheter was defined as a case where permanent catheter insertion cannot be performed normally, such as in patients with abnormal blood flow during HD due to infection or malfunction.

#### 2.2.1. Infection of the Permanent Catheter

Catheter infection occurred when the following cases were suspected or confirmed in patients with permanent catheter insertion:

- When infection clues such as redness, tenderness, heating sense, and pus were suspected at the permanent catheter exit site, and infection was suspected simultaneously in laboratory tests;
- When no evidence of infection before catheter insertion was noted; however, symptoms of infection such as fever emerged within 5 days after insertion, and infection was confirmed on blood culture (no prominent infection was in other organs).

#### 2.2.2. Malfunction of the Permanent Catheter

Catheter malfunction occurred when the following cases were suspected or confirmed in a patient who had inserted a permanent catheter:

- Failure to attain and maintain an extracorporeal blood flow sufficient to perform HD (<300 mL/min);
- Catheter dysfunction due to catheter-tip malposition and mechanical kinking of the catheter.

The CONUT score and GNRI scores were used in the nutrient score and formula below:

CONUT score = serum albumin score + TC score + TLC score. Serum albumin score (0, ≥3.5 g/dL; 2, 3.0–3.49 g/dL; 4, 2.50–2.99 g/dL; 6, <2.50 g/dL), TC score (0, ≥180 mg/dL; 1, 140–180 mg/dL; 2, 100–139 mg/dL; 3, <100 mg/dL), and TLC score (0, ≥1600 103/µL; 1, 1200–1599 103/µL; 2, 800–1199 103/µL; 3, <800 103/µL) [10].

GNRI = 1.489 × serum albumin (g/L) + 41.7 × actual weight (kg)/ideal weight (kg). The ideal weight was calculated using the Lorentzian formula [11,12].

### 2.3. Statistical Analysis

IBM SPSS Statistics version 26 software (IBM Corp., Armonk, NY, USA) was used and *p* value < 0.05 was considered statistically significant. 

A BCM monitor (Fresenius Medical Care, Deutschland GmbH, Germany) was used to evaluate body composition before dialysis on the day of dialysis. In addition, an independent *t*-test was conducted to determine whether each factor had a significant effect on each group. Among them, receiver operating characteristics–area under the curve (ROC-AUC) analysis was used to obtain the best cutoff values for the parameters that showed significant results in the independent *t*-test. 

The best cutoff values obtained through the ROC-AUC curve analysis in the nonpatent group were classified into groups below and above the cutoff values. Continuous use of the permanent catheter was verified using the Kaplan–Meier curve in order to confirm the difference between the groups.

Multivariate Cox regression was adjusted for age, sex, underlying diseases (DM and HTN), and BCM parameters (ECW and ICW) and was performed to verify if the cutoff value of the factor that showed a significant result in the overall group. The adjusted hazard ratio (aHR) at 95% confidence interval (95% CI) was calculated.

## 3. Results

Table 1 summarizes participant characteristics at baseline. The study population included 74 male (44.6%; median age, 81 years) and 56 female (55.4%; median age, 82 years) patients. The patent and nonpatent groups included 103 and 63 patients, respectively. The median age of the patent group was 81 years and that of the nonpatent group was 83 years. The proportion of women was higher in the nonpatent group (69.8%) than in the patent group (46.6%). The nonpatency rates of the dialysis catheter were higher in women (69.8%) than in men (30.2%). Both groups had high rates of HTN as a comorbidity. No statistically significant differences were found in other comorbidities or medication histories. 

Independent sample t-tests were performed to determine the presence of a significant difference between the patent and nonpatent groups in terms of the nutrient score, BCM parameters, and predialysis blood tests (Table 1). As a result, age and sexual differences were significant (*p* < 0.05). Also, CRP and albumin in predialysis blood tests were significant (*p* < 0.05). BCM parameters such as ECW, ECW/ICW ratio, LTI, FTI, LTM, ATM, and BCM did not show significant results (*p* < 0.05); however, ICW showed a *p* value of <0.05. Above all, the nutrient scores such as the CONUT score and GNRI scores showed significant results (*p* < 0.05). More patients required VA creation in the patent group (86.4%) than in the nonpatent group (50.8%), showing a statistically significant difference (*p* < 0.05). The baseline characteristics of the infection and malfunction groups belonging to the nonpatent group are listed in Table 1. 

**Figure 2:**
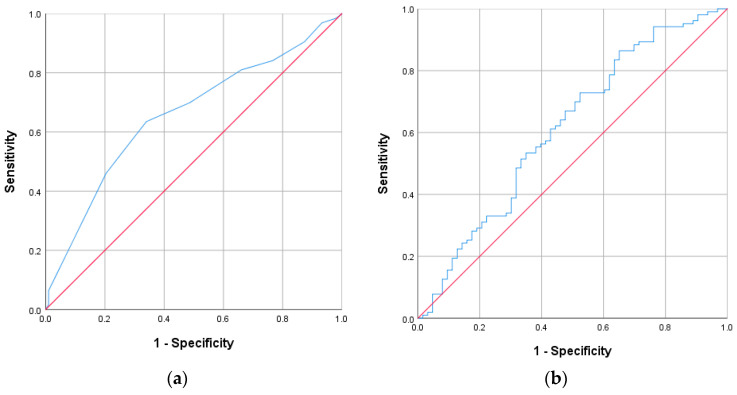
ROC analysis of the CONUT and GNRI scores in the patent and nonpatent groups. (**a**) CONUT score; AUC: 0.657, cutoff value: 6, *p* value: 0.001; (**b**) GNRI score, AUC: 0.613, cutoff value: 90.5, *p* value: 0.015.

**Figure 3:**
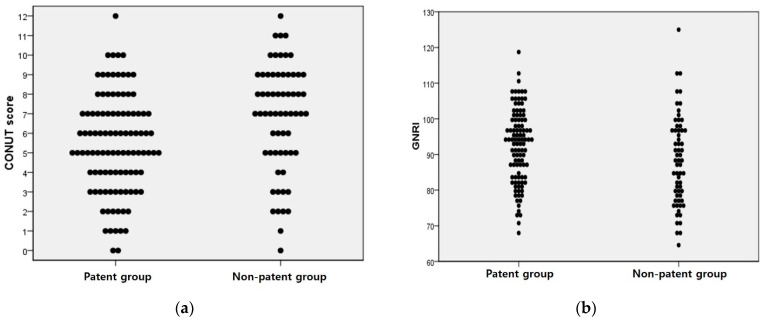
Dot plot with the independent samples *t*-test results of the nutrient scores between groups. (**a**) CONUT score, *p* value: 0.001; (**b**) GNRI score, *p* value: 0.018.

**Figure 4:**
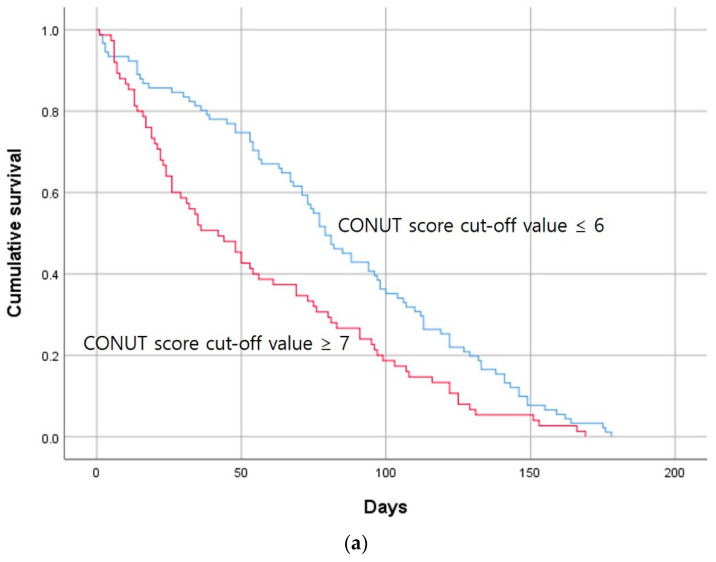

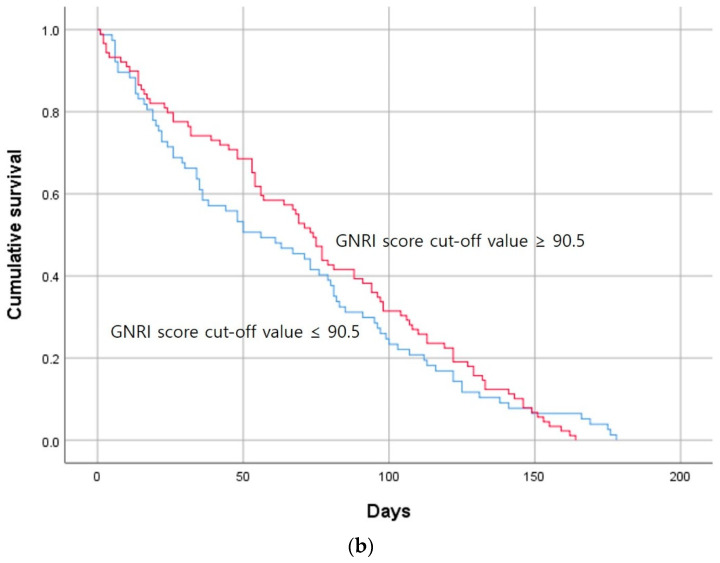
Kaplan–Meier survival curves showing the 180-day permanent catheter survival of the patent and nonpatent groups using the cutoff values obtained for the nutrient score by ROC curve analysis. (**a**) CONUT score (*n* = 166; log-rank χ^2^ = 10.466, *p* = 0.001) and (**b**) GNRI score (*n* = 166; log-rank χ^2^ = 0.231, *p* = 0.231). CONUT, controlling nutritional status; GNRI, geriatric nutritional risk index.

ROC curves were then calculated to confirm the type of association of the CONUT and GNRI scores between the groups (Figure 2). Distribution tables for the nutrient scores are expressed as dot plots to examine the degree of distribution (Figure 3). Based on the cutoff values obtained from the ROC curves, the Kaplan–Meier survival curve was derived within 180 days of survival by dividing it into groups below and above the cutoff values (Figure 4). In the case of the CONUT score, the AUC was confirmed to be 0.657, and after dividing the groups based on the cutoff value of 6 obtained from the ROC-AUC analysis (Figure 2a), the Kaplan–Meier curve showed a significant result with a log–rank *p* value of 0.001 (Figure 4a). The group with a low CONUT score of 6 points or lower showed superior survival in terms of permanent catheter patency. This showed that the patency of the permanent catheter was further reduced in patients with worse nutritional status at the time of permanent catheter insertion. The AUC the GNRI score was 0.613, and when the cutoff value of 90.5 obtained through ROC-AUC analysis was applied to the Kaplan–Meier curve (Figure 2b), the log–rank *p* value showed a nonsignificant result of 0.231 (Figure 4b). This result did not prove the relationship with permanent catheter patency using the GNRI score.

To determine the individual effects of various factors on permanent catheter patency in the nonpatent group, a univariate Cox proportional hazards analysis was performed. (Table 2) Age, CRP, albumin, and CONUT score showed statistically significant results among several factors. (*p* < 0.05) BCM parameters such as LTI and LTM did not show statistically significant results in univariate analysis.

A multivariate Cox proportional hazards analysis was performed to determine whether the CONUT score played an important role in permanent catheter patency among other factors that showed significant results. (Table 3) Based on the independent *t*-test and univariate Cox analysis results (Table 2), factors that showed significant results, comorbidities (DM, HTN), and BCM parameters (ECW and ICW, which are important indicators for volume status) were included step by step in the multivariate Cox analysis. Even when multivariate Cox regression was performed while adding other variables (age, sex, DM, HTN, ECW, and ICW) in stages with models 1, 2, 3, and model 4, the adjusted hazard ratio (aHR) of the CONUT score remained with a high significance (Table 3).

## 4. Discussion

In patients aged >75 years who initiated dialysis using a tunneled dialysis catheter, nutritional status significantly influenced catheter patency. LTI and LTM measured by bioelectrical impedance exhibited lower values in the nonpatent group, although they were not statistically significant. Conversely, ATM and BMI were higher in the nonpatent group; however, no statistically significant results were observed. Stenvinkel et al. reported a counterintuitive relationship between BMI on mortality; patients with a lower BMI exhibited an elevated rate of mortality [13]. Zhou et al. established that high ECW/ICW was linked with a heightened risk of sarcopenia independent of BMI, pre-albumin, C-reactive protein, and other potential confounders in patients with MHD. Notably, this correlation was more prominent in older patients and those with a higher BMI and a history of diabetes [14]. Gracia-lguacel reported that the significant ICW decrease described the loss of LTM and the association of the ICW decrease with the mortality of patients on dialysis, and a negative association between ICW and inflammation and malnutrition [15,16]. The results of the BCM parameters were not statistically significant in this study but showed a similar trend to previous studies [14,15,16] which showed the association with an ICW decrease and inflammation. Kim et al. reported that the parameters ECW/ICW ratio <1.2 and LTI ≥ 10.2 could be useful predictors whether malfunction or infection might affect permanent catheter patency [17]. The study targeted patients who required dialysis and had a permanent catheter insertion at that time. The age of the patient group was 26–93 years old, with a median age of 72 years. This study was conducted only on elderly patients over 75 years of age, and the characteristics of elderly patients were identified to determine factors affecting permanent catheter patency. The patient population in this study was 75 to 97 years old, with a median age of 82 years. In the study published by Kim et al., the study was not conducted only on elderly people over 75 years of age and did not show statistical significance between permanent catheter patency and nutritional status [17]. In the case of elderly patients over 75 years of age, nutritional status, especially CONUT score, was shown to be an important factor affecting permanent catheter patency. Significant findings were noted for albumin and inflammation markers. The CONUT and GNRI scores, reflecting albumin levels, demonstrated statistically significant differences between the two groups. Among them, the CONUT score remained a statistically significant factor in the multivariate Cox proportional hazards analysis considering other covariates. One of the primary causes of functional failure in tunneled HD catheters is infection. B. Tanriover et al. demonstrated a significantly increased risk of infection in patients with hypoalbuminemia compared with those with normal serum albumin levels [18]. Nathan W. Levin et al. stated that albumin is an important indicator of the survival rate of patients on dialysis and is associated with inflammation. A decrease in albumin concentration may be linked to an increase in inflammatory markers [19]. Suat Unver et al. reported a positive correlation between hypoalbuminemia and bacteremia. Hypoalbuminemia was identified as a risk factor for the occurrence of catheter-related bacteremia; therefore, improving hypoalbuminemia should be considered to reduce infectious complications [20]. Albumin synthesis is downregulated because of decreased nutrient intake; therefore, low albumin levels are generally used as an indicator of PEW. In addition, synthesis is also downregulated in inflammatory states. Therefore, low albumin levels are considered both a marker of acute factors in protein status and an indicator of inflammation [21]. The inflammatory response cascade activates leukocytes, which subsequently secrete myeloperoxidase, a catalyst in the formation of platelet aggregates. This process can lead to the development of a friable clot at or within the lumen of the catheter tip, potentially culminating in catheter dysfunction [22,23]. Malnutrition arises from an intricate web of causative factors. Beyond hypoalbuminemia, an inflammatory response incites the release of growth hormones, glucagon, cortisol, and catecholamines, precipitating insulin resistance and fuel-store mobilization. The activation of interleukin and tumor necrosis factor-alpha serves to accelerate the catabolism of muscle protein, whereas interleukin-6 enhances the creation of acute phase proteins via the liver. Indicators such as serum albumin, pre-albumin, C-reactive protein, and alpha-1 acid glycoprotein are recognized as universal markers of stress, independent of the underlying cause [24]. When correlated with the results of high CRP levels and low albumin levels in the nonpatent group than the patent group in this study, lower LTI and LTM by bioelectrical impedance were presumed to be caused by muscle protein breakdown due to these inflammatory markers. The proportion of patients who abandoned vascular access was higher in the nonpatent group. Kaller et al. reported the predictive role of inflammatory indicators and the importance of vascular diameter in AVF maturation failure [25]. Other studies have examined the relationship between systemic inflammation and AVF failure [26,27]. In this study, the failure to create vascular access in the group with high inflammation markers and relatively low nutritional status was consistent with the results of previous studies. Several national and international guidelines recommend both physical and nutritional therapy for patients undergoing the procedure to improve their experience and outcomes [28,29,30]. When considering these factors together, prevention through early identification of patients on HD with malnutrition, along with appropriate therapeutic interventions, are important management methods to consider to improve not only the patient experience but also the patency of the dialysis catheter [31,32,33].

This study has several limitations. First, this is a single-institution retrospective review that relies on available patient information; therefore, it may contain potentially incomplete or missing information, selection bias, and an inability to establish causal relationships. During the recruitment of the study group targeting patients aged >75 years, many cases of follow-up loss were noted among patients with relatively good dialysis catheter function after being transferred to a nursing hospital. Thus, the generalization of our results to another population may not be valid. Second, the scope of racial or ethnic diversity among study participants was not examined, as the sample was exclusively composed of South Korean individuals. Therefore, corroborative studies conducted across different countries or healthcare institutions that demonstrate significant impacts of nutrient scores on catheter patency would substantiate these findings more conclusively. Third, although adjustments were made for various covariates, the potential for residual confounding by unmeasured or unconsidered variables persists, which may have affected the study’s outcomes. Moreover, due to the retrospective design and the urgent clinical requirements of the participants for permanent catheterization for dialysis, conducting a comprehensive evaluation for sarcopenia was unfortunately not feasible. Future research endeavors that encompass multinational and multicenter cohorts, alongside strategies to mitigate the attrition of patients transferred to nursing facilities, would enhance the inclusivity of the study population. Such advancements are anticipated to surmount the current study’s constraints and yield more dependable findings. Further investigations examining the persistence of permanent catheter patency relative to nutrient scores will be invaluable. Specifically, discerning any interrelations between sarcopenia and racial disparities could significantly inform the clinical management of patients receiving maintenance hemodialysis (HD).

## 5. Conclusions

Nutritional status evaluated by CONUT scores measured before the start of dialysis are expected to play an important role to predicting the prognosis of permanent catheter-patency survival in patients aged 75 years or older. Specifically, identifying and managing a group of patients with very poor nutrient scores is expected to be of great help in improving the treatment and prognosis of patients on maintenance HD. Additional long-term prospective studies in other cohorts are required to confirm the usefulness of the nutrient scores proposed in this study as important predictors of permanent catheter-patency survival. Catheter-patency studies according to the nutrient scores are needed when dialysis is initiated in medical centers in other countries where various races can be treated. This method is expected to be of great help in the management of patients who maintain dialysis treatment through a permanent catheter.

## Figures and Tables

**Table 1:** Baseline characteristics.

Characteristic	Overall (*N* = 166)	Catheter Group		*p*
		Patent Group (*N* = 103)	Nonpatent Group (*N* = 63)	
Age (median [range] years)	82.0 [75–97]	81.0 [75–97]	83.0 [75–92]	0.016
Sex [*n* (%)]				
Men	74 (44.6)	55 (53.4)	19 (30.2)	0.003
Women	92 (55.4)	48 (46.6)	44 (69.8)	
Comorbidities [*n* (%)]				
Diabetes mellitus	93 (56.0)	62 (60.2)	31 (49.2)	0.168
Hypertension	141 (84.9)	85 (82.5)	56 (88.9)	0.269
Heart failure	67 (40.4)	42 (40.8)	25 (39.7)	0.890
Ischemic heart disease	45 (27.1)	32 (31.1)	13 (20.6)	0.144
Atrial fibrillation	41 (24.7)	23 (22.3)	18 (28.6)	0.369
Cerebral infarction	22 (13.3)	17 (16.5)	5 (7.9)	0.115
Liver cirrhosis	11 (6.6)	3 (2.9)	8 (12.7)	0.014
Medication [*n* (%)]				
Aspirin	50 (30.1)	28 (27.2)	22 (34.9)	0.295
Clopidogrel	36 (21.7)	23 (22.3)	13 (20.6)	0.799
Warfarin	9 (5.4)	6 (5.8)	3 (4.8)	0.771
Cilostazol	15 (9.0)	12 (11.7)	3 (4.8)	0.135
NOAC	12 (7.2)	11 (10.7)	1 (1.6)	0.028
Statin	70 (42.2)	48 (46.6)	22 (34.9)	0.141
Laboratory data (median [range])				
Hemoglobin (g/dL)	9.7 [5.5–13.2]	9.7 [5.5–13.2]	9.5 [6.3–11.9]	0.685
Total lymphocyte count (10^3^/µL)	1100.0 [120–4620]	1160.0 [120–3700]	940.0 [130–4620]	0.233
Platelet (000s)	166.0 [12–520]	165.0 [12–445]	140.0 [20–520]	0.224
CRP (mg/dL)	2.5 [0.1–40]	1.4 [0.1–40.0]	6.8 [0.2–37.8]	<0.001
Total protein (g/dL)	6.0 [3.9–7.9]	6.1 [4.2–7.6]	5.9 [3.9–7.9]	0.340
Albumin (g/dL)	3.0 [1.6–4.3]	3.1 [2.2–4.3]	2.8 [1.6–4.1]	<0.001
BUN (mg/dL)	51.9 [13.0–184.5]	53.3 [13.0–184.5]	51.0 [17.4–118.0]	0.708
Creatinine (mg/dL)	4.3 [0.5–16.2]	4.5 [0.7–16.2]	3.8 [0.5–10.8]	0.051
Total cholesterol (mg/dL)	127.5 [50–290]	129.0 [50–239]	121.0 [50–290]	0.526
Total calcium (mg/dL)	8.0 [5.5–11.1]	8.0 [5.5–10.2]	8.0 [5.7–11.1]	0.292
Phosphorus (mg/dL)	4.0 [0.3 –10.7]	4.1 [1.2–10.7]	3.9 [0.3–7.4]	0.175
Sodium (mEq/L)	138.0 [121–153.6]	138.0 [126–147.0]	137.8 [121–153.6]	0.821
Potassium (mEq/L)	4.4 [2.8–7.8]	4.5 [2.8–7.0]	4.3 [2.8–7.8]	
Body composition (median [range])				
ECW (L)	13.9 [7.8–31.1]	14.4 [7.8–31.1]	13.7 [8.4–23.7]	0.137
ICW (L)	13.3 [7.9–30.4]	14.3 [7.9–30.4]	11.9 [8.2–20.7]	0.012
TBW (L)	27.3 [16.3–53.5]	28.4 [16.3–53.5]	26.0 [18.3–43.7]	0.028
ECW/ICW ratio	1.10 [0.53–1.90]	1.07 [0.53–1.90]	1.13 [0.75–1.56]	0.101
LTI (kg/m^2^)	10.3 [5.3–26.1]	11.3 [5.8–26.1]	9.7 [5.3–23.3]	0.089
FTI (kg/m^2^)	10.8 [0.1–33.8]	10.2 [0.1–33.8]	11.2 [4.3–25.0]	0.180
LTM (kg)	25.16 [2.2–69.4]	27.6 [2.2–69.4]	23.2 [13.8–49.4]	0.097
ATM (kg)	24.9 [0.2–56.2]	24.2 [0.2–52.2]	25.1 [2.4–56.2]	0.162
FAT (kg)	19.0 [0.3–41.3]	18.3 [0.3–38.4]	19.1 [7.2–41.3]	0.133
Dry weight (kg)	52.8 [24.8–92.5]	53.3 [24.8–89.3]	51.5 [32.8–92.5]	0.641
BMI (kg/m^2^)	23.6 [15.1–44.3]	23.5 [15.1–34.2]	23.8 [15.3–44.3]	0.255
Information on initiating hemodialysis (median [range])				
Ultrafiltration volume (L)	1.5 [0.2–4.0]	1.5 [0.3–4.0]	1.5 [0.2–4.0]	0.268
Further management [*n* (%)]				
VA creation	121 (72.9)	89 (86.4)	32 (50.8)	<0.001
VA abandonment	45 (27.1)	14 (13.6)	31 (50.2)	
Nutrient score (median [range])				
CONUT score	6.0 [0–12]	5.0 [0–12]	7.0 [0–12]	0.001
GNRI score	91.5 [64.6–125.0]	93.7 [68.0–118.7]	87.8 [64.6–125.0]	0.018
Permanent catheter use date (median [range])	69.0 [1–178]	88.0 [6–178]	63.0 [1–141]	<0.001

ATM, adipose-tissue mass; BMI, body mass index; BUN, blood urea nitrogen; CONUT, controlling nutritional status; CRP, C-reactive protein; ECW, extracellular water; FAT, fat mass; VA, vascular access; FTI, fat-tissue index; GNRI, geriatric nutritional risk index; ICW, intracellular water; LTI, lean-tissue index; LTM, lean-tissue mass; NOAC, nonvitamin K antagonist oral anticoagulant; TBW, total body water.

**Table 2:** Univariate Cox proportional hazards analysis of the permanent catheter patency in the nonpatent group.

	Hazard Ratio	95% CI of the Difference		*p* Value
		Lower	Upper	
Sex	0.828	0.609	1.126	0.229
Age	1.048	1.013	1.085	0.007
DM	0.876	0.642	1.194	0.402
HTN	0.901	0.587	1.383	0.634
BMI	1.029	0.996	1.063	0.084
ECW	0.997	0.961	1.034	0.871
ICW	0.980	0.943	1.019	0.311
TBW	0.994	0.974	1.014	0.528
E/I ratio	1.869	0.905	3.861	0.091
LTI	0.971	0.930	1.015	0.197
FTI	1.030	0.999	1.062	0.055
LTM	0.995	0.981	1.009	0.494
ATM	1.011	0.997	1.025	0.121
FAT	1.015	0.996	1.035	0.115
Hemoglobin	0.956	0.856	1.067	0.421
Total lymphocyte count	1.000	1.000	1.000	0.131
Platelet	0.998	0.996	1.000	0.071
CRP	1.049	1.027	1.071	<0.001
Total protein	0.962	0.785	1.179	0.708
Albumin	0.664	0.485	0.910	0.011
BUN	0.999	0.993	1.006	0.865
Creatinine	0.959	0.900	1.021	0.190
Total cholesterol	0.999	0.995	1.002	0.467
Total calcium	1.042	0.869	1.249	0.656
Phosphorus	0.930	0.827	1.045	0.221
Sodium	1.001	0.968	1.036	0.936
Potassium	0.965	0.805	1.156	0.698
CONUT score	1.655	1.213	2.259	0.001
GNRI score	0.927	0.678	1.267	0.634

**Table 3:** Multivariate Cox proportional hazards analysis of the permanent catheter patency in the nonpatent group.

	Hazard Ratio	95% CI of the Difference		*p* Value
		Lower	Upper	
Model 1	1.655	1.213	2.259	0.001
Model 2	1.665	1.215	2.281	0.002
Model 3	1.690	1.232	2.319	0.001
Model 4	1.714	1.234	2.381	0.001

CI, confidence interval; CONUT, controlling nutritional status; ECW, extracellular water; ICW, intracellular water. Model 1: adjusted for the CONUT score. Model 2: adjusted for the covariates included model 1, age, and sex. Model 3: adjusted for the covariates included model 2 and comorbidities (DM and HTN). Model 4: adjusted for the covariates included Model 3 and BCM parameters (ECW and ICW).

## Data Availability

The original contributions presented in the study are included in the article; further inquiries can be directed to the corresponding authors.
